# Supplementary material for: Inflammatory factors and risk of lung adenocarcinoma: a Mendelian randomization study mediated by blood metabolites
Source: Front Endocrinol (Lausanne). 2024 Aug 27;15:1446863. doi: 10.3389/fendo.2024.1446863 (PMC11384989; doi:10.3389/fendo.2024.1446863)
Supplement: Supplementary file 1 [file DataSheet1.zip › supplementary files/S2/GCST90200519/sensitivity-analysis.pdf]

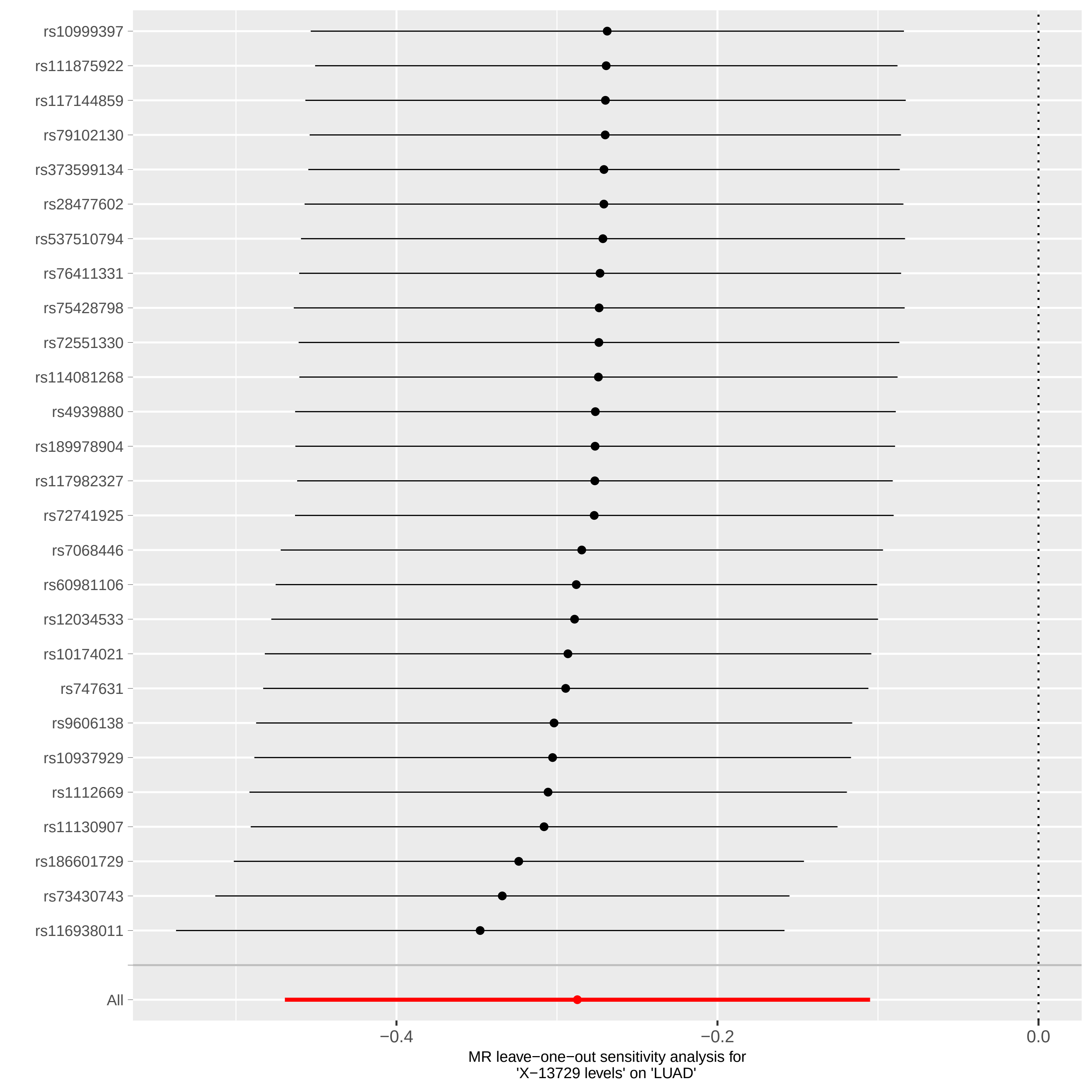

rs10999397
rs111875922
rs117144859
rs79102130
rs373599134
rs28477602
rs537510794
rs76411331
rs75428798
rs72551330
rs114081268
rs4939880
rs189978904
rs117982327
rs72741925
rs7068446
rs60981106
rs12034533
rs10174021
rs747631
rs9606138
rs10937929
rs1112669
rs11130907
rs186601729
rs73430743
rs116938011
All
−0.4
−0.2
0.0
MR leave−one−out sensitivity analysis for
'X−13729 levels' on 'LUAD'
